# Novel Genetic Findings in a Chinese Family with Axenfeld-Rieger Syndrome

**DOI:** 10.1155/2017/5078079

**Published:** 2017-06-13

**Authors:** Kuanshu Li, Liu Yang, Ying Liu, Ding Lin

**Affiliations:** ^1^Aier School of Ophthalmology, Central South University, Changsha, China; ^2^Aier Eye Hospital, Changsha, Hunan, China

## Abstract

**Purpose:**

To describe a Chinese family with Axenfeld-Rieger syndrome (ARS) and report our novel genetic findings.

**Methods:**

Nine members of the same family underwent complete ophthalmologic examinations and genetic analysis. Genomic DNA was isolated from veinal blood and amplifed using PCR; the products of PCR were sequenced and compared with FOXC1 and PITX2 genes, from which the mutations were found.

**Results:**

Through the ophthalmologic examinations, 8 subjects were diagnosed as ARS and 1 subject was normal. A homozygous mutation c.1139_1141dupGCG(p.Gly380_Ala381insGly) and a heterozygous mutation c.1359_1361dupCGG(p.Gly456_Gln457insGly) in FOXC1 were identified in all subjects. The mutation (c.-10-30T>C) was identified in PITX2 in subjects III-1 and III-3.

**Conclusions:**

We found novel gene mutations in a Chinese family with ARS, which provides us with a better understanding of the gene mutation spectrum of ARS and the assistance for the genetic counseling and gene-specific therapy in the future.

## 1. Introduction

Axenfeld-Rieger syndrome (ARS) is a rare autosomal dominant disorder, characterized by anterior segment abnormalities and systemic abnormalities [1, 2]. Common anterior segment abnormalities include [3–5] iris hypoplasia, corectopia, polycoria, iridocorneal adhesions, posterior embryotoxon, and glaucoma. The systemic abnormalities include [1, 6] the cardiovascular outflow tract, midface hypoplasia, flat nasal root, maxillary and mandibular hypoplasia, hypertelorism and telecanthus, skeletal anomalies, hearing loss, dental abnormalities, and redundant periumblical skin. ARS has complete penetrance but variable expressivity [7].

To date, two major genes, forkhead box C1 (*FOXC1*) on chromosome 6p25 and pituitary homeobox 2 (*PITX2*) on chromosome 4q25, have been demonstrated to cause ARS. Mutations in FOXC1 and PITX2 can explain about 40% of ARS [1, 8, 9]. Mutations in CYP1B1 was identified in a child with ARS and congenital glaucoma [10]. Micheal et al. reported an ARS family caused by mutations in PRDM5 [11]. Riise et al. reported the association between ARS and *PAX6* deletion [12], but then it was found to be incorrect [13]. In addition, two other loci on chromosomes 13q14 and 16q24 have been suggested to be associated with ARS by linkage analyses, but the specific disease-causing genes have not yet been identified [4, 14].

In this study, we performed complete ophthalmologic examinations and analysis of FOXC1 and PITX2 of all subjects and report our novel genetic findings.

## 2. Materials and Methods

### 2.1. Subjects

**Figure 1:**
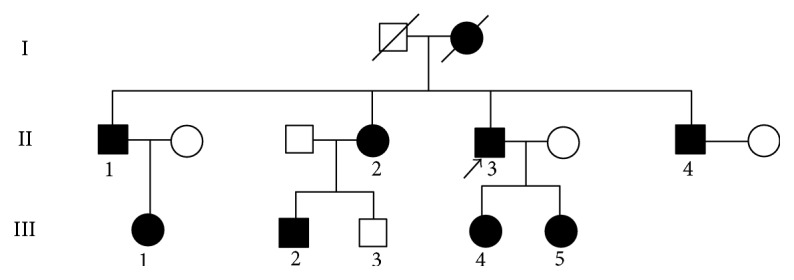
Pedigree of the family with ARS. Arrow signals the proband. Squares indicate males, and circles indicate females. Black symbols represent affected individuals, and white symbols represent unaffected individuals.

Two generations of a Chinese family with ARS were recruited to Aier Eye Hospital of Changsha (Figure 1). The study was approved by the ethics committee of Aier Eye Hospital of Changsha and adhered to the tenets of the Declaration of Helsinki. We received informed consent from all subjects before the study.

### 2.2. Clinical Evaluations

We performed full ophthalmologic examinations on all subjects, including visual acuity, intraocular pressure measurements (Goldman), slit lamp, anterior segment photography, visual field test (Humphrey 750, Carl Zeiss, Germany), and anterior segment OCT (Carl Zeiss, Germany). If the refractive medium was clear, we also performed funduscopy and gonioscopic and retinal nerve fiber layer (RNFL) thickness measurements (Carl Zeiss, Germany).

### 2.3. Mutation Analysis

About 2 ml of venous blood was sampled from each subject and collected in vacutainer tubes (Sanjiu Medical Technology Co., Ltd., Liuyang, China) containing EDTA. Genomic DNA was extracted from each blood using a genomic DNA mini kit for blood (Life Technologies). All coding exons, with flanking intronic regions, of *FOXC1* and *PITX2* were amplified using PCR with primers. The amplifed DNA was purifed by agarose gel electrophoresis and sequenced on a 3730/3700xl automated DNA sequencer (Applied Biosystems).

## 3. Results

### 3.1. Clinical Evaluations

Through the ophthalmologic examinations, subject III-3 was normal and the other 8 subjects were diagnosed as ARS.

### 3.2. Subject II-3

**Figure 2:**
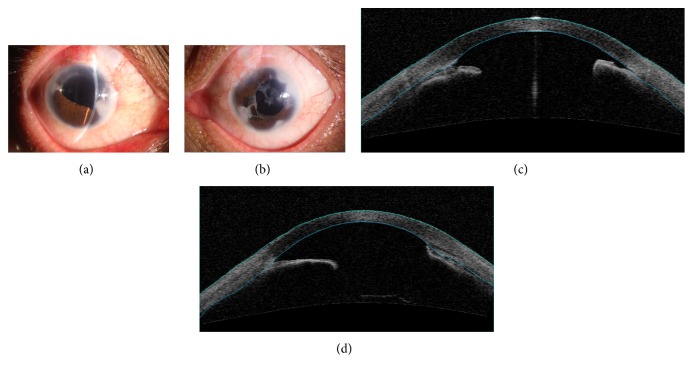
Ocular characteristics of subject II-3. Anterior segment photography showed iris hypoplasia, iridocoloboma, corectopia, and peripheral anterior synechia in both eyes (a, b). Anterior segment OCT showed iridocorneal adhesions in both eyes (c, d).

The proband of this family is male who is 48 years old. He was referred to our hospital because of decreased visual acuity in his right eye. He underwent trabeculectomy and cataract surgery in both of his eyes at another hospital about 10 years ago; however, he had been completely blinded in his left eye. His IOP measured with Goldmann tonometry in the right eye was up to 40 mmHg. Slit lamp examination revealed iris hypoplasia, iridocoloboma, corectopia, and peripheral anterior synechia (Figure 2). No systemic abnormalities was found. We performed trabeculectomy with mitomycin C in his right eye. Postoperatively, the IOP was well controlled.

### 3.3. Subject III-1

**Figure 3:**
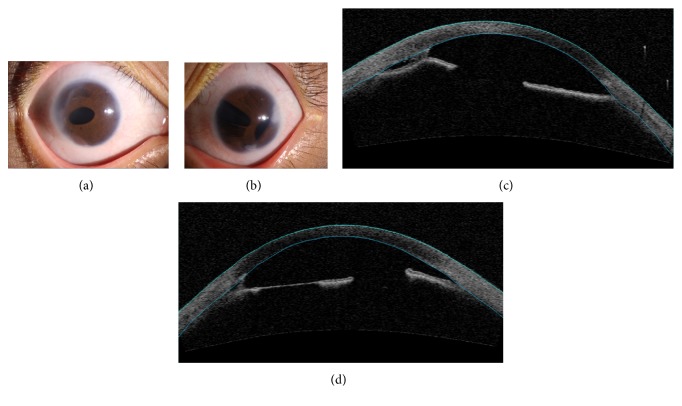
Ocular characteristics of subject III-1. Anterior segment photography showed corectopia and peripheral anterior synechia in the right eye (a) and iris hypoplasia, corectopia, polycoria, and peripheral anterior synechia in the left eye (b). Anterior segment OCT showed iridocorneal adhesions in the right eye (c) and iris hypoplasia and iridocorneal adhesions in the left eye (d).

The proband's niece is female who is 28 years old. She had no history of surgery in her both eyes. At present, she uses timolol, azopt, and alphagan to control IOP. Her IOP measured with Goldmann tonometry was 35 mmHg in the right eye and 36 mmHg in the left eye. Corectopia and peripheral anterior synechia were seen in the right eye, and iris hypoplasia, corectopia, polycoria, and peripheral anterior synechia were seen in the left eye (Figure 3). No systemic abnormalities was found. We advised her to have antiglaucoma surgery in both her eyes as soon as possible.

### 3.4. Subject III-3

**Figure 4:**
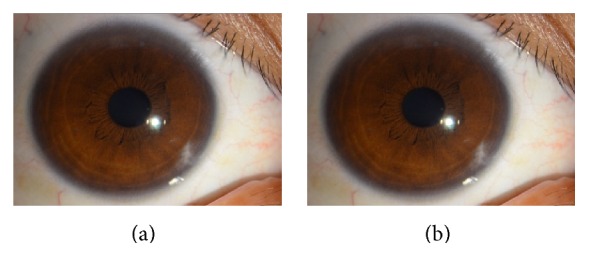
Ocular characteristics of subject III-3. Anterior segment photography showed no anterior segment abnormality in both eyes (a, b).

The proband's nephew is male who is 31 years old. His uncorrected visual acuity was 20/20 in both eyes. IOP measured by Goldmann tonometry was 10 mmHg in the right eye and 12 mmHg in the left eye. Slit lamp examination revealed no anterior segment abnormalities (Figure 4).

The visual field test was normal. No systemic abnormalities was found.

### 3.5. Genetic Analysis of PITX2 and FOXC1

**Figure 5:**
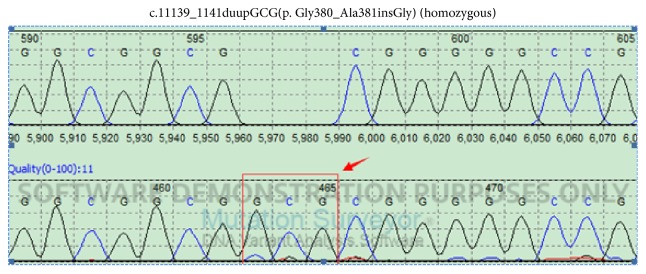
DNA sequence analysis of FOXC1 shows a homozygous mutation c.1139_1141dupGCG(p.Gly380_Ala381insGly) in all subjects.

**Figure 6:**
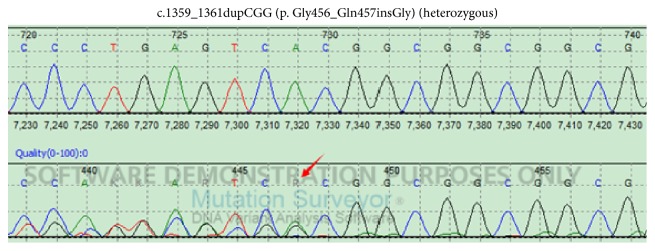
DNA sequence analysis of FOXC1 shows a heterozygous mutation c.1359_1361dupCGG(p.Gly456_Gln457insGly) in all subjects.

DNA sequence analysis of FOXC1 shows a homozygous mutation c.1139_1141dupGCG(p.Gly380_Ala381insGly) (Figure 5) and a heterozygous mutation c.1359_1361dupCGG(p.Gly456_Gln457insGly) (Figure 6) in all subjects.

**Figure 7:**
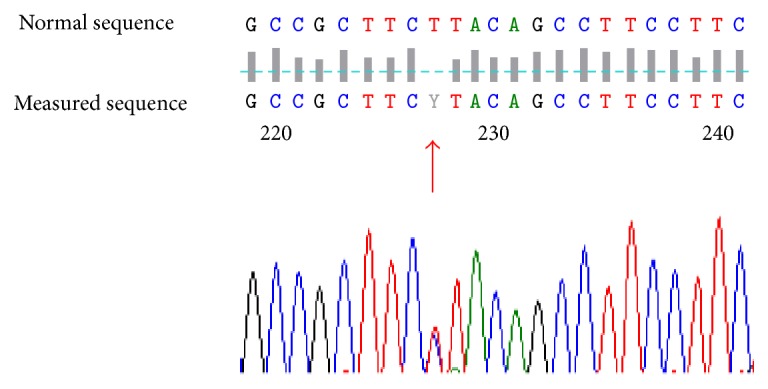
DNA sequence analysis of PITX2 shows a heterozygous mutation *c.-10-30T>C* in subjects III-1 and III-3.

DNA sequence analysis of PITX2 shows a heterozygous mutation *c.-10-30T>C* in subjects III-1 and III-3 (Figure 7).

## 4. Discussion

Axenfeld-Rieger syndrome is a rare autosomal dominant disorder where phenotypes of the same mutation are variable; this is likely to be caused by environmental factors and/or modifier genes [15]. In our study, a homozygous mutation c.1139_1141dupGCG(p.Gly380_Ala381insGly) and a heterozygous mutation c.1359_1361dupCGG(p.Gly456_Gln457insGly) in FOXC1 were found in all subjects, but their clinical findings were variable. The proband showed iris hypoplasia, corectopia, and peripheral anterior synechia; subject III-1 showed corectopia and peripheral anterior synechia in the right eye and iris hypoplasia, corectopia, polycoria, and peripheral anterior synechia in the left eye, even subject III-3 showed no abnormities. This is consistent with previous reports [16, 17].

The two major genes of ARS are FOXC1 and PITX2. FOXC1 is a member of the forkhead family of transcription factors, which recognizes and binds to specific DNA sequences through the conserved 110-amino-acid forkhead domain (FH) and thus activates the target genes [18–20].

FOXC1 is strongly expressed in the skeletal muscle, kidney, liver, and heart and plays important roles in embryogenesis, tissue-specific gene expression, and tumor development [20, 21]. Mutations in FOXC1 include intragenic mutations, microscopic and submicroscopic deletions, and duplications [22]. PITX2 is a member of the bicoid-like homeobox transcription factor family, which plays important roles in the genetic control of development, particularly in pattern formation and the determination of cell fate [8, 23]. *PITX2* consists of six exons and encodes a bicoid-like homeodomain transcription factor involved in embryogenesis [9]. Its expression in neural crest cells is necessary for the development of optic stalk and anterior segment [24]. PITX2 mutations include intragenic mutations, microscopic and submicroscopic deletions, and chromosome rearrangements such as translocations [22].

In our study, a homozygous mutation c.1139_1141dupGCG(p.Gly380_Ala381insGly) and a heterozygous mutation c.1359_1361dupCGG(p.Gly456_Gln457insGly) were identified in FOXC1 in all subjects. But the homozygous mutation is in the ClinVar database as rs398123611 where it is listed as a benign variant. Similarly, the heterozygous mutation is also in the ClinVar as re398123612 where it is listed as a benign/likely benign variant. The point our research deserves special attention to is that the mutation (c.-10-30T>C), which was identified in PITX2 in subjects III-1 and III-3, has not been reported before. Most of the intragenic *PITX2* mutations are loss-of-function mutations, which result in defective DNA binding or/and decreased transactivation capability of downstream genes [4]. Both PITX2 and FOXC1 are dosage sensitive; the alteration in the level of functional protein (either increased or decreased) is a mechanism of the disease.

The major clinical concern of ARS is glaucoma, which caused serious damage to eyesight, and glaucoma may occur in about 50% patients with ARS [4, 25]; the severity of glaucoma correlates with the level of iris inserting into the angle [26]. But only 18% of the patients with ARS responded to medical or surgical (used solely or in combination) treatment, this may be due to surgical complications such as early fibrosis and the presence of modifier genes [27]. In our study, except subject III-3 and subject III-5, the rest all had glaucoma; however, the severities were differential. Subject II-1, subject II-2, subject II-3, and subject II-4 were close to blind, and subject III-2 and subject III-4 were well controlled with medicine, while subject III-1 cannot be controlled with medicine.

In summary, we described variable clinical findings in a Chinese family with ARS in this report. They showed variable phenotypes and had no systemic abnormalities. We performed DNA sequence of FOXC1 and PITX2; a homozygous mutation c.1139_1141dupGCG(p.Gly380_Ala381insGly) and a heterozygous mutation c.1359_1361dupCGG(p.Gly456_Gln457insGly) in FOXC1 were identified in all subjects. The mutation (c.-10-30T>C) was identified in PITX2 in subjects III-1 and III-3. Though more functional studies are still needed to prove the association between these mutations and ARS, our results are useful for a better understanding of the spectrum of *FOXC1* and *PITX2* mutations and provide help for the genetic counseling and gene-specific therapy in the future.
